# Nigramide J is a novel potent inverse agonist of the human constitutive androstane receptor

**DOI:** 10.1002/prp2.18

**Published:** 2014-01-26

**Authors:** Yuichiro Kanno, Nobuaki Tanuma, Tomofumi Yatsu, Wei Li, Kazuo Koike, Yoshio Inouye

**Affiliations:** Faculty of Pharmaceutical Sciences, Toho UniversityMiyama 2-2-1, Funabashi, Chiba, 274-8510, Japan

**Keywords:** Constitutive androstane receptor, inverse agonist, nigramide, nuclear receptor

## Abstract

The constitutive androstane receptor (CAR, NR1I3) is very important for drug development and for understanding pharmacokinetic drug–drug interactions. We screened by mammalian one hybrid assay among natural compounds to discover novel ligands of human constitutive androstane receptor (hCAR). hCAR transcriptional activity was measured by luciferase assay and mRNA levels of CYP2B6 and CYP3A4 in HepTR-hCAR cells and human primary hepatocytes were measured by real-time RT-PCR. Nigramide J (NJ) whose efficacy is comparable to those of hitherto known inverse agonists such as clotrimazole, PK11195, and ethinylestradiol. NJ is a naturally occurring cyclohexane-type amide alkaloid that was isolated from the roots of *Piper nigrum*. The suppressive effect of NJ on the CAR-dependent transcriptional activity was found to be species specific, in the descending order of hCAR, rat CAR, and mouse CAR. The unliganded hCAR-dependent transactivation of reporter and endogenous genes was suppressed by NJ at concentrations higher than 5 *μ*mol/L. The ligand-binding cavity of hCAR was shared by NJ and CITCO, because they were competitive in the binding to hCAR. NJ interfered with the interaction of hCAR with coactivator SRC-1, but not with its interaction with the corepressor NCoR1. Furthermore, NJ is agonist of human pregnane X receptor (hPXR). NJ is a dual ligand of hCAR and hPXR, being an agonist of hPXR and an inverse agonist of hCAR.

## Introduction

The constitutive androstane receptor (CAR, NR1I3) and pregnane X receptor (PXR, NR1I2) are two important members of the nuclear receptor superfamily, playing a key role in response to xenochemical stimuli. The functions of CAR and PXR are also very important for drug development and for understanding pharmacokinetic drug–drug interactions, because they regulate the expression of various drug-metabolizing enzymes and transporters, such as CYP2B, CYP3A, CYP2C, glutathione S-transferases, sulfotransferases, UDP-glucuronosyltransferase 1A1, OATP2, MRP2, and MRP3 (Honkakoski et al. 1998; Sueyoshi et al. 1999; Sugatani et al. 2001; Ferguson et al. 2002; Goodwin et al. 2002; Kast et al. 2002). Recently, CAR has received renewed attention as a molecular target for the treatment of metabolic diseases, because of its key role in energy homeostasis such as thyroid hormone metabolism (Maglich et al. 2004; Qatanani et al. 2005), glucogenesis (Ueda et al. 2002; Kodama et al. 2004; Miao et al. 2006), and lipogenesis (Roth et al. 2008a,b). Furthermore, in vivo studies using ob/ob mice and high-fat diet-fed mice have shown that CAR activation suppresses glucose production, stimulates glucose uptake, improves glucose tolerance and insulin sensitivity, and prevents obesity (Gao et al. 2009; Dong et al. 2009).

The CAR is normally sequestered into the cytoplasmic compartment of untreated liver cells and is translocated to the nucleus after exposure to an activator or ligand. Following nuclear translocation, CAR binds to response elements in the promoter regions of its target genes, forming a heterodimer complex with retinoid X receptor alpha, NR2B1 (Baes et al. 1994). In contrast, exogenously expressed CAR accumulates in the nucleus without any stimulus in cultured cell lines (Kawamoto et al. 1999; Kanno et al. 2005a,b; Kanno et al. 2007). Because CAR has a constitutive transactivation potential, nuclear CAR induces target gene transcription in the absence of agonists (referred to as basal or constitutive activity; Baes et al. 1994). The basal activity may be further enhanced in the presence of agonists. The compounds that repress the basal activity of a ligand-free nuclear receptor are inverse agonists, which are represented by androstenol (Forman et al. 1998), clotrimazole (CLT) (Moore et al. 2000), PK11195 (Li et al. 2008), S07662 (Kublbeck et al. 2011), and ethinylestradiol (EE2) (Jyrkkarinne et al. 2005). Given the species-specific differences, however, the number of inverse agonists of human CAR (hCAR) is fewer; for example the androstane metabolite, androstenol, is an inverse agonist of mouse CAR (mCAR), but its activity is much weaker on hCAR (Forman et al. 1998). To our knowledge, only PK11195 and EE2 have been reported as species-specific inverse agonists of hCAR.

Given our quite limited knowledge on the inverse agonists of CAR, this study set out to identify novel potent inverse agonist toward hCAR from natural resources. By a screening method for hCAR ligands (agonists and inverse agonists) using mammalian one-hybrid assay (Kanno and Inouye 2010), we identified nigramide J (NJ), a naturally occurring cyclohexane-type amide alkaloid, as a novel inverse agonist of hCAR, with more potent activity than any other known inverse agonists. Its modes of action and profiles were consequently investigated in detail.

## Materials and Methods

### Chemicals

NJ used in this study was previously isolated from the roots of *Piper nigrum*. The purity was found to be >98% by HPLC and NMR analyses.

### Plasmids

The expression plasmids for GAL4/DBD-fused hCAR/LBD (GAL4/DBD-hCAR/LBD) and GAL4/DBD-hCAR/LBD(+3a.a.) were constructed as previously reported (Kanno and Inouye 2010). The segment of hPXR/LBD (140-431a.a.) was cloned into pcDNA5-GAL4/DBD. Segments of the receptor interaction domains of SRC-1 (SRC-1/RID, 569–781) and the nuclear receptor corepressor NCoR1 (NCoR1/RID, 1574–2338) were cloned into pcDNA5-GAL4/DBD (Kanno et al. 2013). The preparation of the PBREM-driven luciferase reporter plasmid (PBREM-Luc) has been described elsewhere (Kanno et al. 2005a,b). A DNA fragment encoding hCAR was amplified from pcDNA-hCAR, and cloned into pcDNA5-VP16.

### Cell culture

The HepTR/hCAR cell line was established in human hepatoma HepG2 cells using the T-REx system (Invitrogen, Carlsbad, CA). In this cell line, hCAR was expressed only in the presence of tetracycline (Tet) (Kanno et al. 2012). HepG2 and HepTR/hCAR cells were cultured in Dulbecco's modified Eagle's medium (Wako, Osaka, Japan) containing 10% fetal bovine serum and penicillin-streptomycin in a humidified atmosphere containing 5% CO_2_ at 37°C.

### Luciferase reporter analysis

Using PEI Max reagent (Polysciences Inc., Warrington, PA), cells were transfected with the appropriate expression and reporter plasmids, and with pGL4.74 (hRluc/TK; Promega, Madison, WI) as an internal standard. After overnight incubation, the cells were treated with individual compounds and solvent control [0.1% dimethyl sulfoxide (DMSO)] for 24 h. Luciferase activity was measured using the Dual-Luciferase Reporter Assay System (Promega). The firefly luciferase activity was normalized against that of Renilla luciferase. Primary screening for hCAR ligands was conducted using a single luciferase reporter assay as follows: cells were transfected with pcDNA-GAL4-hCAR/LBD or pcDNA-GAL4-hCAR/LBD(+3a.a.) and pG5luc reporter plasmids.

### Quantitative reverse transcription-PCR

Total RNA was isolated from whole cell lysates using ISOGEN II (Nippon Gene Co., Tokyo, Japan), and cDNA was synthesized using a ReverTraAce qPCR RT Kit (Toyobo, Osaka, Japan). Quantitative reverse transcription-PCR (qRT-PCR) was conducted using a THUNDERBIRD™ SYBR qPCR Mix (Toyobo) according to the manufacturer's protocol and the 7500 fast system SDS software (Applied Biosystems, Carlsbad, CA). Specific primers were used to target *CYP2B6* (5′-AAG CGG ATT TGT CTT GGT GAA-3′ and 5′-TGG AGG ATG GTG GTG AAG AAG-3′), *CYP3A4* (5′-CCAAGCTATGCTCTTCACCG-3′, and 5′-TCAGGCTCCACTTACGGTGC-3′), and β-actin (5′-TCC TCC TGA GCG CAA GTA CTC-3′ and 5′-CTG CTT GCT GAT CCA CAT CTG-3′).

### Mammalian two-hybrid assay

HepG2 cells were transfected with the pG5luc reporter plasmid (0.1 *μ*g), the VP16-hCAR expression plasmid (0.05 *μ*g), pGL4.74 (0.01 *μ*g), and the expression plasmid for GAL4-SRC-1/RID or GAL4-NCoR1/RID (0.05 *μ*g) using PEI Max reagent. The following day, the cells were treated with NJ or solvent control [0.1% DMSO] for 24 h. Luciferase activity was measured using the Dual-Luciferase Reporter Assay System.

### Human primary hepatocytes and treatment

Human primary hepatocytes (BIOPREDIC International, Rennes, France) were plated and cultured according to the manufacturer's protocol. Cells were plated on 24-well collagen-coated culture plates with Seeding Medium MIL212. After 6 h, the medium was exchanged for Incubation Medium MIL214. The next day, cells were treated with each compound for 24 h. The mRNA expression level was measured by the abovementioned qRT-PCR.

### Statistical analysis

Statistically significant differences were determined using the one-way ANOVA analysis followed by Tukey's multiple comparison test as the post hoc test, and differences were considered statistically significant at *P* < 0.05.

## Results

### Discovery of NJ as an hCAR inverse agonist

**Figure 1:**
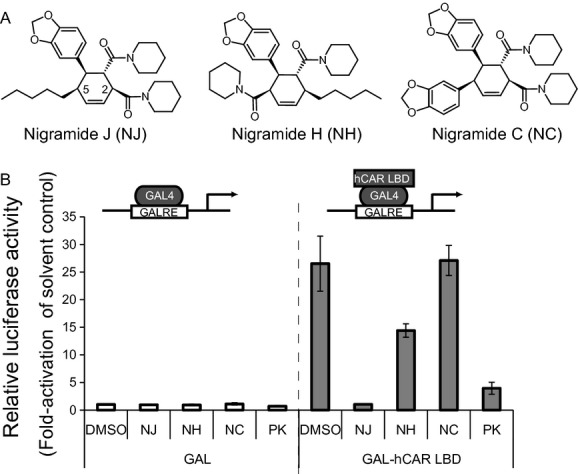
Effect of NJ on the transcriptional activity of human constitutive androstane receptor (hCAR). (A) Chemical structures of NJ, NC, and NH. (B) HepG2 cells were transfected with the pG5luc reporter plasmid (0.1 *μ*g) together with the expression vectors for GAL4/DBD (0.05 *μ*g) or GAL4/DBD-hCAR/LBD (0.05 *μ*g) and pGL4.74 (0.01 *μ*g) plasmids. Cells were treated with NJ, NC, and NH (10 *μ*mol/L) or a positive control [PK11195 (10 *μ*mol/L)]. After 24 h, luciferase activity was measured using the Dual-Luciferase Reporter Assay System. Results are shown as fold activation over the solvent control of GAL4/DBD empty plasmid (mean ± SD, *n* = 3).

Screening for hCAR ligands was carried out using the assay system established in our previous study (Kanno and Inouye 2010). The mammalian one-hybrid assay using GAL4/DBD fused to hCAR/LBD or hCAR/LBD(+3a.a.) was employed to distinguish between agonists and inverse agonists of hCAR in HepG2 cells, which were simultaneously transfected with GAL4RE-driven luciferase reporter plasmids. By screening a natural compound library using this system, we identified NJ showing a potent suppression of the GAL4/DBD-hCAR/LBD-dependent basal transactivation that was comparable to that of PK11195 (Fig. 1B). These preliminary data suggested that NJ may be a candidate potent inverse agonist of hCAR of natural compound origin. However, structure-related cyclohexane-type amide alkaloids from the same plants (Wei et al. 2005a) and chemical synthesized analogs (Wei et al. 2005b) did not show this activity, as exemplified by nigramide H (NJ) and nigramide C (NC) (Fig. 1A).

### Dose-dependent effect of NJ on the hCAR-dependent expression of a reporter gene in HepG2 cells

**Figure 2:**
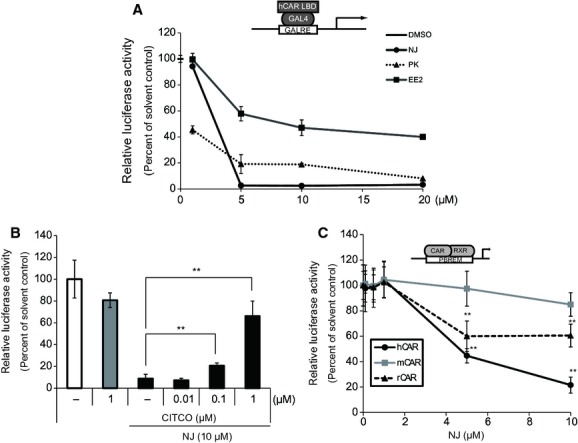
Identification of NJ as an inverse agonist of human constitutive androstane receptor (hCAR). (A, B) HepG2 cells were transfected with an expression vector for GAL4/DBD-hCAR/LBD (0.05 *μ*g) in the presence of the pG5luc (0.1 *μ*g) and pGL4.74 (0.01 *μ*g) plasmids. The transfected cells were treated with (A) increasing concentrations of NJ, PK, EE2 (1, 5, 10, 20 *μ*mol/L) or solvent control (0.1% DMSO) (B) CITCO alone (1 *μ*mol/L) or NJ (10 *μ*mol/L) alone, NJ (10 *μ*mol/L) plus CITCO (0.01, 0.1 or 1 *μ*mol/L). After 24 h, luciferase activity was measured using the Dual-Luciferase Reporter Assay System. Results are shown as percentage of basal activity of hCAR (solvent control) and the activity of GAL4 empty vector is set to zero(mean ± SD, *n* = 3); **P < 0.01. (C) HepG2 cells were transfected with an expression vector for human, mouse, or rat CAR (0.05 *μ*g) in the presence of the PBREM-luc (0.1 *μ*g) and pGL4.74 (0.01 *μ*g) plasmids. Results are shown as percentage of basal activity of CAR (solvent control) and the activity of GAL4 empty vector is set to zero (mean ± SD, *n* = 4).

To verify the suppressive effect of NJ on the basal transcriptional activity of hCAR, HepG2 cells transfected with GAL4/DBD-hCAR/LBD were treated with increasing concentrations of NJ. hCAR-dependent basal transactivation showed the maximum decrease at a NJ concentration of 5 *μ*mol/L (Fig. 2A). We compared the suppressive effect of NJ with those of two known inverse agonists, PK11195 and EE2. As shown in Figure 2A, at equimolar concentrations the suppressive effect of NJ can be considered as at least as potent as PK11195 or EE2.

Then, we investigated whether the CAR agonist, CITCO, can reverse the NJ-mediated repression. Suppression of CAR activity by NJ was reversed by co-treatment with CITCO in a concentration-dependent manner (Fig. 2B). These results suggest that NJ is a novel inverse agonist of hCAR.

### Species-specific effect of NJ on the CAR-dependent expression of a reporter gene in HepG2 cells

Because some CAR ligands have been reported to be species specific, we compared the effect of NJ on the transactivation of a reporter gene depending on hCAR, mCAR, or rat CAR (rCAR). HepG2 cells were transfected with the PBREM-reporter plasmid and the hCAR, mCAR, or rCAR expression plasmids (Fig. 2C). The most remarkable repression of reporter gene expression was observed with hCAR. A less significant repression was observed with rCAR, while no effect was observed with mCAR.

### Effect of NJ on the hCAR-dependent expression of endogenous genes in HepTR/hCAR cells

**Figure 3:**
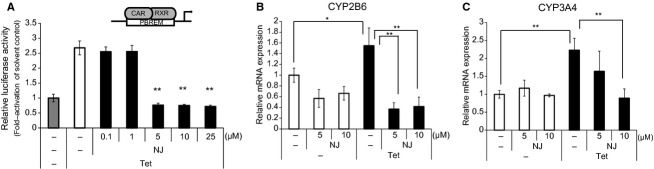
Effects of NJ on the expression of endogenous CAR target genes in HepTR/human constitutive androstane receptor (hCAR) cells. (A) HepTR/hCAR cells were transfected with the PBREM-luc and pGL4.74 plasmids. The transfected cells were treated with increasing concentrations of NJ (0.1, 1, 5, 10, 25 *μ*mol/L) and solvent control (0.1% DMSO) in the absence or presence of Tet. After 24 h, luciferase activity was measured using the Dual-Luciferase Reporter Assay System. Results are shown as fold activation over the solvent control (mean ± SD, *n* = 4); **P < 0.01. (B, C) HepTR/hCAR cells were treated with NJ (5 or 10 *μ*mol/L) or solvent control (0.1% DMSO) in the absence or presence of Tet. After 48 h, cells were harvested and the mRNA level of *CYP2B6* (B) and *CYP3A4* (C) was measured by real-time qRT-PCR. Results normalized against β-actin mRNA levels are expressed as fold activation over the solvent control (mean ± SD, *n* = 4); *P < 0.05; **P < 0.01.

Next, we examined the effect of NJ on the expression of endogenous hCAR target genes in HepTR/hCAR cells, in which hCAR was only expressed in the presence of Tet. As shown in Figure 3A, the Tet-induced PBREM-luciferase reporter activity was repressed by NJ in HepTR/hCAR cells as well as in transient experiments. Tet treatment upregulated the mRNA levels of the hCAR target genes, *CYP2B6* and *CYP3A4* (Fig. 3B,C). Furthermore, Tet-mediated induction of *CYP2B6* and *CYP3A4* mRNA was reduced by co-treatment with NJ.

### Effect of NJ on the interactions between hCAR and its coregulators (coactivator or corepressor)

**Figure 4:**
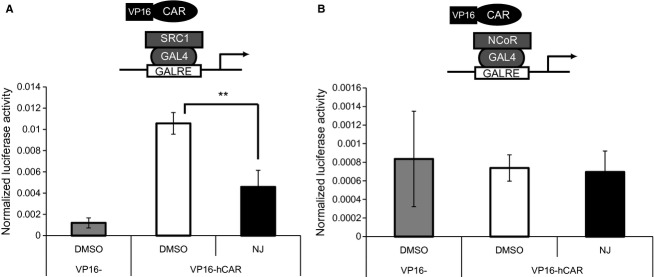
Effect of NJ on the interaction between human constitutive androstane receptor (hCAR) and its cofactors. HepG2 cells were transfected with pG5luc (0.1 *μ*g) and pGL4.74 (0.01 *μ*g) reporter plasmids, and with the expression plasmids for VP16-hCAR and GAL4-SRC-1/RID (A) or GAL4-NCoR1/RID (B). After 24 h, cells were treated with NJ (10 *μ*mol/L) or solvent control (0.1% DMSO). Luciferase activity was measured 24 h post-treatment. Results are presented as Renilla-normalized luciferase activity (mean ± SD, *n* = 4); **P < 0.01.

It is known that the suppression of hCAR transactivation by inverse agonists is mediated by dissociation of coactivators and/or association of corepressors. To determine whether NJ alters cofactor recruitment by hCAR, a mammalian two-hybrid assay was carried out in HepG2 cells transfected with pG5luc, VP16-hCAR, or VP16, and GAL4-SRC-1/RID or GAL4-NCoR1/RID. NJ repressed the interaction of hCAR with SRC-1/RID (Fig. 4A). Although PK11195 and TO901317 increased interaction between hCAR and NCoR1/RID (Kanno et al. 2013), NJ did not affect the interaction (Fig. 4B).

### Effect of NJ on the hPXR-dependent expression of a reporter gene in HepG2 cells

**Figure 5:**
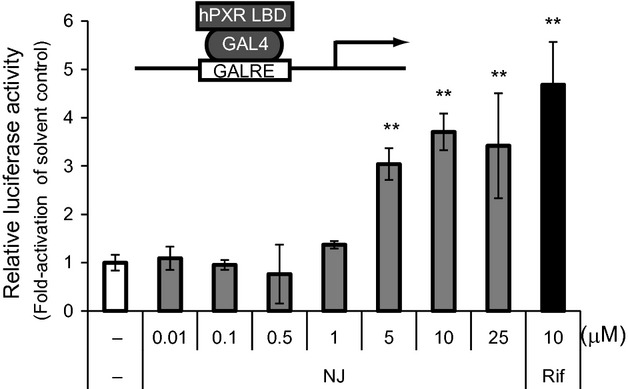
Regulation of the transcriptional activity of hPXR by NJ. HepG2 cells were transfected with an expression vector for GAL4/DBD-hPXR/LBD (0.05 *μ*g) together with the pG5luc (0.1 *μ*g) and pGL4.74 (0.01 *μ*g) plasmids. The transfected cells were treated with the solvent control (0.1% DMSO) or increasing concentrations of NJ (0.01, 0.1, 0.5, 1, 5, 10, 25 *μ*mol/L) or positive control [Rifampicin (Rif, 10 *μ*mol/L)]. After 24 h, luciferase activity was measured using the Dual-Luciferase Reporter Assay System. Results are expressed as fold activation over the solvent control (mean ± SD, *n* = 4); **P < 0.01.

Almost all known inverse agonists of hCAR also act as hPXR agonists. To test whether NJ could activate hPXR, we carried out a luciferase reporter assay using GAL4/DBD-hPXR/LBD. As shown in Figure 5, hPXR transcriptional activity increased upon NJ treatment in a dose-dependent manner. This result shows that NJ is a dual ligand of hCAR and hPXR.

### Effect of NJ on the hCAR-dependent expression of endogenous genes in human primary hepatocytes

**Figure 6:**
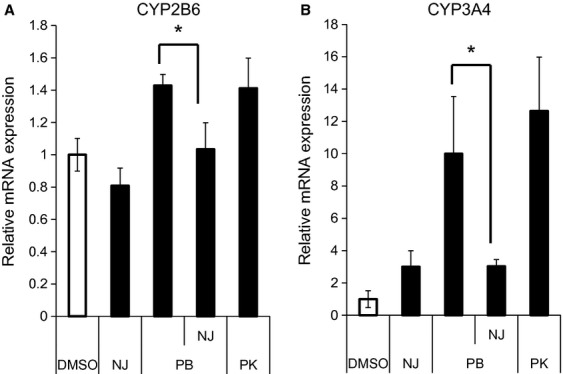
Effects of NJ on the expression of human constitutive androstane receptor target genes in human primary hepatocytes. Human primary hepatocytes were treated with NJ (10 μmol/L), PK11195 (10 μmol/L), or a solvent control (0.1% DMSO) in the absence or presence of phenobarbital (PB, 1 mmol/L). After 24 h, cells were harvested and the mRNA levels of *CYP2B6* (A) and *CYP3A4* (B) were measured by real-time qRT-PCR. The results are from four replicates and results normalized against β-actin mRNA levels are presented as fold expression over the solvent control (mean ± SD, *n* = 4). *P < 0.05.

Finally, we tested whether NJ could attenuate hCAR-dependent *CYP3A4* and *CYP2B6* mRNA induction in human primary hepatocytes. As shown in Figure 6, treatment with CAR activator phenobarbital (PB) resulted in an increase in about 1.4-fold and 10-fold in *CYP2B6* and *CYP3A4* mRNA levels, respectively. Co-treatment with NJ attenuated both PB-induced *CYP2B6* and *CYP3A4* mRNA expression. In the absence of PB, NJ induced a slight increase in *CYP3A4* mRNA (threefold) expression, while it slightly repressed the expression of CYP2B6.

## Discussion

Both CAR and PXR (SXR) play key roles in drug sensing and mobilization of drug-metabolizing cascades by regulating the expression of drug-metabolizing enzymes represented by cytochrome P450s, such as CYP2B6, CYP2C9, and CYP3A4. Unlike PXR and other nuclear receptors, CAR expressed in the nuclear compartment of cultured cell lines retains basal (or constitutive) transcriptional activity in the absence of any ligands. On the basis of this phenomenon, CAR ligands are further subdivided into agonists and inverse agonists. In this study, NJ was found to be a novel potent inverse agonist of hCAR. NJ is a natural occurring cyclohexane-type amide alkaloid, isolated from the roots of *Piper nigrum* (Wei et al. 2005a,b), though the physiological and/or biological functions of this compound have not been elucidated, yet.

Only a few inverse agonists of hCAR have been reported previously. In this study, NJ caused significant deactivation similar to known potent inverse agonist of hCAR, EE2, and PK11195 in HepG2 cells (Fig. 2A). Concerning the species specificity (Fig. 2C), the marked inverse agonist activity of NJ on hCAR was not observed with rCAR and mCAR. Because this assay was using human HepG2-derived heterodimer partner RXR and cofactors, further assessment in rodent cells may be required. Similarly, the inverse agonist nature of PK11195 observed with hCAR was not observed with mCAR (Li et al. 2008).

Given the rather limited number of known NJ structural analogs, structure–activity considerations remain highly preliminary. Some of the previously reported known cyclohexane-type amide alkaloids did not show such inverse agonist activity as NJ did. Inactive nigramide C is differentiated from active NJ only by the existence of 3,4-methylenedioxyphenyl in C-5 of the cyclohexene ring, suggesting that the C-5 alkane substitution is essential for the potent inverse agonist activity. Nigramide H, which has reverse substitutions in C-2 and C-5 compared with NJ, exhibited weak activity.

To investigate the effect of NJ on the hCAR-dependent expression of endogenous genes, we used HepTR/hCAR cells, in which hCAR was only expressed in the presence of Tet. NJ completely inhibited the hCAR (or Tet)-dependent expression of the endogenous target genes, *CYP2B6* and *CYP3A4*, in HepTR/hCAR cells. Most of the known inverse agonists of CAR also act as agonists of PXR, for example, PK11195, S07662, and CLT are known as potent PXR agonists (Moore et al. 2000; Li et al. 2008; Kublbeck et al. 2011). Here, we showed that NJ could also activate PXR (Fig. 5). HepTR/hCAR cells expressed very low levels of PXR, because the induction of *CYP3A4* was hardly observed after treatment with the potent PXR agonist, rifampicin (data not shown). Thus, the repression of the expression of *CYP2B6* and *CYP3A4* can be attributed to functional hCAR. Furthermore, we observed NJ-mediated repression of PB-induced *CYP3A4* and *CYP2B6* mRNA expression in human primary hepatocytes (Fig. 6). Previous reports have shown that PK11195 and S07662, dually acting as CAR inverse agonists and PXR agonists, induced *CYP3A4* mRNA expression in human primary hepatocytes (Li et al. 2008; Kublbeck et al. 2011). In this study, we also observed the potent induction of *CYP3A4* by PK11195 in human primary hepatocytes, in agreement with these previous reports. Interestingly, only a slight induction of *CYP3A4* expression was observed after NJ treatment compared with that after PK11195 treatment. This observation suggests that NJ is a novel type dual ligand of hCAR and PXR.

CAR-dependent transactivation in the nucleus requires interactions with cofactors. Because CAR has constitutive transactivity, unliganded CAR can interact with coactivators such as SRC-1, TIF2, and PGC-1 (Muangmoonchai et al. 2001; Min et al. 2002; Shiraki et al. 2003). This study demonstrated that NJ could interfere with the interaction between hCAR and SRC-1 in a two-hybrid assay. In contrast, the interaction between hCAR and the corepressor NCoR1 was not affected by NJ treatment. These results suggest that NJ mainly mediates the suppression of hCAR transactivity by dissociating the coactivator, SRC-1. However, it is possible that NJ disrupts the interaction between other corepressor or coactivator and hCAR.

In conclusion, we have disclosed a novel inverse agonist of hCAR, NJ, which is classified into a different structural category from the known hCAR inverse agonists, and has the most potent inverse agonist activity among hCAR inverse agonists hitherto known. The discovery of NJ as a potent inverse agonist of hCAR will provide further insight into the molecular basis of hCAR function.

## Abbreviations

CAR: constitutive androstane receptor
CLT: clotrimazole
EE2: ethinylestradiol
NJ: nigramide J
PB: phenobarbital
PXR: pregnane X receptor
qRT-PXR: Quantitative reverse transcription-PCR
Tet: tetracycline
